# Heat Shock Protein 70 Prevents Hyperoxia-Induced Disruption of Lung Endothelial Barrier via Caspase-Dependent and AIF-Dependent Pathways

**DOI:** 10.1371/journal.pone.0129343

**Published:** 2015-06-11

**Authors:** Dmitry Kondrikov, David Fulton, Zheng Dong, Yunchao Su

**Affiliations:** 1 Department of Pharmacology & Toxicology, Medical College of Georgia, Georgia Regents University, Augusta, GA 30912, United States of America; 2 Department of Medicine, Medical College of Georgia, Georgia Regents University, Augusta, GA 30912, United States of America; 3 Department of Cell Biology and Anatomy, Medical College of Georgia, Georgia Regents University, Augusta, GA 30912, United States of America; 4 Vascular Biology Center, Medical College of Georgia, Georgia Regents University, Augusta, GA 30912, United States of America; 5 Research Service, Charlie Norwood Veterans Affairs Medical Center, Augusta, Georgia 30912, United States of America; University of Illinois College of Medicine, UNITED STATES

## Abstract

Exposure of pulmonary artery endothelial cells (PAECs) to hyperoxia results in a compromise in endothelial monolayer integrity, an increase in caspase-3 activity, and nuclear translocation of apoptosis-inducing factor (AIF), a marker of caspase-independent apoptosis. In an endeavor to identify proteins involved in hyperoxic endothelial injury, we found that the protein expression of heat-shock protein 70 (Hsp70) was increased in hyperoxic PAECs. The hyperoxia-induced Hsp70 protein expression is from hspA1B gene. Neither inhibition nor overexpression of Hsp70 affected the first phase barrier disruption of endothelial monolayer. Nevertheless, inhibition of Hsp70 by using the Hsp70 inhibitor KNK437 or knock down Hsp70 using siRNA exaggerated and overexpression of Hsp70 prevented the second phase disruption of lung endothelial integrity. Moreover, inhibition of Hsp70 exacerbated and overexpression of Hsp70 prevented hyperoxia-induced apoptosis, caspase-3 activation, and increase in nuclear AIF protein level in PAECs. Furthermore, we found that Hsp70 interacted with AIF in the cytosol in hyperoxic PAECs. Inhibition of Hsp70/AIF association by KNK437 correlated with increased nuclear AIF level and apoptosis in KNK437-treated PAECs. Finally, the ROS scavenger NAC prevented the hyperoxia-induced increase in Hsp70 expression and reduced the interaction of Hsp70 with AIF in hyperoxic PAECs. Together, these data indicate that increased expression of Hsp70 plays a protective role against hyperoxia-induced lung endothelial barrier disruption through caspase-dependent and AIF-dependent apoptotic pathways. Association of Hsp70 with AIF prevents AIF nuclear translocation, contributing to the protective effect of Hsp70 on hyperoxia-induced endothelial apoptosis. The hyperoxia-induced increase in Hsp70 expression and Hsp70/AIF interaction is contributed to ROS formation.

## Introduction

Prolonged exposure to increased concentrations of oxygen induces diffuse pulmonary injuries, vascular leakage, excessive inflammation, and pulmonary edema [1,2]. Lung vascular endothelial alterations represent the most striking pathophysiological changes in hyperoxia-induced lung injuries [3,4]. Several studies have confirmed that exposure of lung endothelial cells to high concentrations of oxygen causes the disruption of endothelial barrier [5–7]. Hyperoxia exposure leads to oxidative stress resulting in lung endothelial injury [4,8]. Increased generation of reaction oxygen species (ROS) and reactive nitrogen species leads to protein oxidation and nitration and causes lung endothelial damage including barrier disruption and cell death [4,8–10]. At the same time, various protective mechanisms are deployed to prevent cells from hyperoxia-induced oxidative stress and cell damage [11,12]. For example, increased expression of p21(Cip1) during hyperoxia delays the loss of the anti-apoptotic proteins Mcl-1 and Bcl-X(L) [11]. Alphonse et al. reported that increased Akt activation reduces hyperoxia-induced apoptosis and preserves alveolar architecture in the experimental bronchopulmonary dysplasia [12].

In an endeavor to identify proteins involved in hyperoxic lung injury, we found that protein expression of heat shock protein 70 (Hsp70) is increased in pulmonary artery endothelial cells (PAECs) exposed to hyperoxia. Hsp70 is a member of a molecular chaperone family involved in repairing misfolded proteins that occurs as a result of various extracellular stresses including heat, mechanical damage, and hypoxia [13,14]. Hsp70 is involved in the down-regulation of NOX1 and NOX2 activity and plays an important role in the protection from ROS-induced vascular dysfunction [15]. Nevertheless, it remains unknown how Hsp70 is regulated at hyperoxic condition and what the role of Hsp70 is in hyperoxic lung endothelial barrier disruption. In the present study, we found that Hsp70 protects against endothelial barrier disruption through caspase-dependent and apoptosis-inducing factor (AIF)-dependent mechanisms. Hyperoxia-induced increase in Hsp70 expression is caused by increased ROS formation. Manipulation of Hsp70 might be a novel therapy for acute lung injury in oxygen toxicity.

## Materials and Methods

### Reagents and materials

Mouse anti-Hsp70 antibody was obtained from Transduction Laboratory (Lexington, KY). Anti-β-actin monoclonal antibody was from Sigma (St. Louis, MO). Anti-cleaved caspase-3 antibody was from Cell Signaling (Boston, MA). AIF antibody was from Santa Cruz (Santa Cruz, CA). The Hsp70 inhibitor KNK437 and the caspase inhibitor-I Z-VAD-FMK were from EMD Millipore (Billerica, MA). N-acetylcystein (NAC) and other reagents were purchased from Sigma (St. Louis, MO) unless specified otherwise.

### Cell culture and hyperoxic exposure

Bovine PAECs were obtained from ATCC (Manassas, VA). Cells were kept in F12K medium supplemented with 10% FBS and antibiotics solutions (Corning CellGro, Manassas, VA). Cells of passages two to ten were used for all experiments. For hyperoxic exposure, confluent PAECs were exposed to normoxia (room air, 5% CO_2_) or to hyperoxia (95% oxygen, 5% CO_2_) in the oxygen chamber for 1 to 24 h or 48 h at 1 atmosphere.

### Determination of Hsp70 mRNA and protein levels

Total mRNA from PAECs was isolated using RNeasy Mini Kit (Qiagen, Valencia, CA) according to manufacturer’s instruction. Bovine Hsp70 hspA1A, hspA1B and hspA2 primers were obtained from Life Technologies (Grand Island, NY). Reverse transcription was performed using High Capacity cDNA Reverse Transcription kit (Applied Biosystem). Quantitative real-time PCR was performed using CYBR green Master Mix from BioRad Laboratories (Hercules, CA) on a Step One Plus real time PCR system (Life Technologies, Grand Island, NY) according to manufacturer instruction. Hsp70 protein level was measured using Western blot and quantified by densitometry using Quantity One 1-D analysis software.

#### Endothelial monolayer permeability assay

ECIS-Zeta electric cell-substrate impedance sensing system was used to determine transendothelial electrical resistance (TEER) value as a measurement of endothelial barrier integrity. Confluent endothelial cells plated in gold-coated electrodes arrays (Applied Biophysics, Troy, NY). After resistances were relatively constant (1000–1200 Ω), treatments (the Hsp70 inhibitor KNK437, the caspase-3 inhibitor Z-VAD-FMK and NAC) were added directly to the wells and arrays were allowed to equilibrate for another 3 h. Cells then were exposed to 95% oxygen, 5% CO_2_ for up to 72 h. The impedance was recorded for the duration of the experiments. Data were normalized to the mean resistance before hyperoxia exposure.

### Overexpression and silencing of Hsp70

PAEC were incubated with sham adenovirus containing red fluorescence protein (RFP) or adenovirus containing human hspA1A gene, which encodes human Hsp70 protein, at MOI of 5.0 for 1 h [15]. F12K medium containing 10% FBS then was added to reach final concentration of 4% FBS. After incubation for 48 h, cells were then exposed to hyperoxia or normoxia before being used for TUNEL and endothelial monolayer permeability assay. Hsp70 expression was silenced using siRNA technology. Hsp70 siRNA and negative control siRNA were from Santa Cruz (Dallas, TX). Hsp70 siRNA is a mixture of siRNAs against mRNAs of hspA1A and hspA1B. The siRNAs were transfected into PAECs with siPORT amine transfection reagent according to the manufacturer’s instruction. 3 days after transfection, the medium was changed to serum-free medium for 24 h followed by the treatments of PAECs.

### Apoptosis assays

TUNEL staining and caspase-3 assay were used to detect apoptosis. For TUNEL assay, PAECs were plated on LabTek 8 well chamber slides (Thermo Fisher, Waltham, MA). After exposure to hyperoxia for 48 h apoptosis was detected using ApopTag Plus Fluorescent in situ Apoptosis Detection Kit (Millipore, Temecula, CA, USA) according manufacturer’s protocol. Nuclei were stained with DAPI. Slides were evaluated by Zeiss LSM 510 laser scanning confocal microscope using filters for FITC and DAPI (excitation/emission 490/520 nm and 358/461 nm, respectively). For caspase-3 assay, confluent PAECs were exposed to 95% oxygen for 48 h and then washed with ice-cold PBS and lysed in lysis buffer. Caspase-3 activity was measured using a colorimetric assay kit (R&D systems, Minneapolis, MN, USA) according to manufacturer’s protocol. The enzymatic reaction was performed in 96-well plates at 37°C using caspase-3 colorimetric substrate, DEV-pNA. Caspase activity was assessed by Spectra Max M2 micro-plate reader (Molecular Devices Sunnyvale, CA).

### AIF nuclear translocation assay

Nuclear fractions of hyperoxia-exposed PAECs were isolated using NE-PER Nuclear Extraction Reagent Kit (Thermo Scientific, Rockford, IL) according to manufacturer’s protocol. Lysates were subjected to Western blot using AIF antibody.

### Co-immunoprecipitation of AIF and Hsp70

The lysates of cytosolic fraction of PAECs exposed to normoxia or hyperoxia were incubated with anti-AIF antibody or non-immune IgG at 4°C overnight. 30 μl of protein-A sepharose was added and samples were further incubated for 2 h at 4°C. Immunoprecipitates were collected by centrifugation ad washed three times in buffer containing 50 mM Tris-HCl pH 7.5, 150 mM NaCl, and 0.1% Triton X-100. Proteins were eluted from sepharose beads by boiling the samples in 30 μl of SDS sample buffer. Sepharose beads were pelleted by centrifugation and supernatants were analyzed by Western blot using appropriate antibodies.

### Statistical analysis

Within each experiment, cells were matched for number of passages to avoid differences related to tissue culture variables. Results are shown as means ± SE for *n* experiments. One way ANOVA and *t* test analysis (2-tailed) were used to determine the significance of differences between the means of experimental and control groups. A value of *P* < 0.05 was considered significant.

## Results

### Hyperoxia increases Hsp70 expression

**Fig 1:**
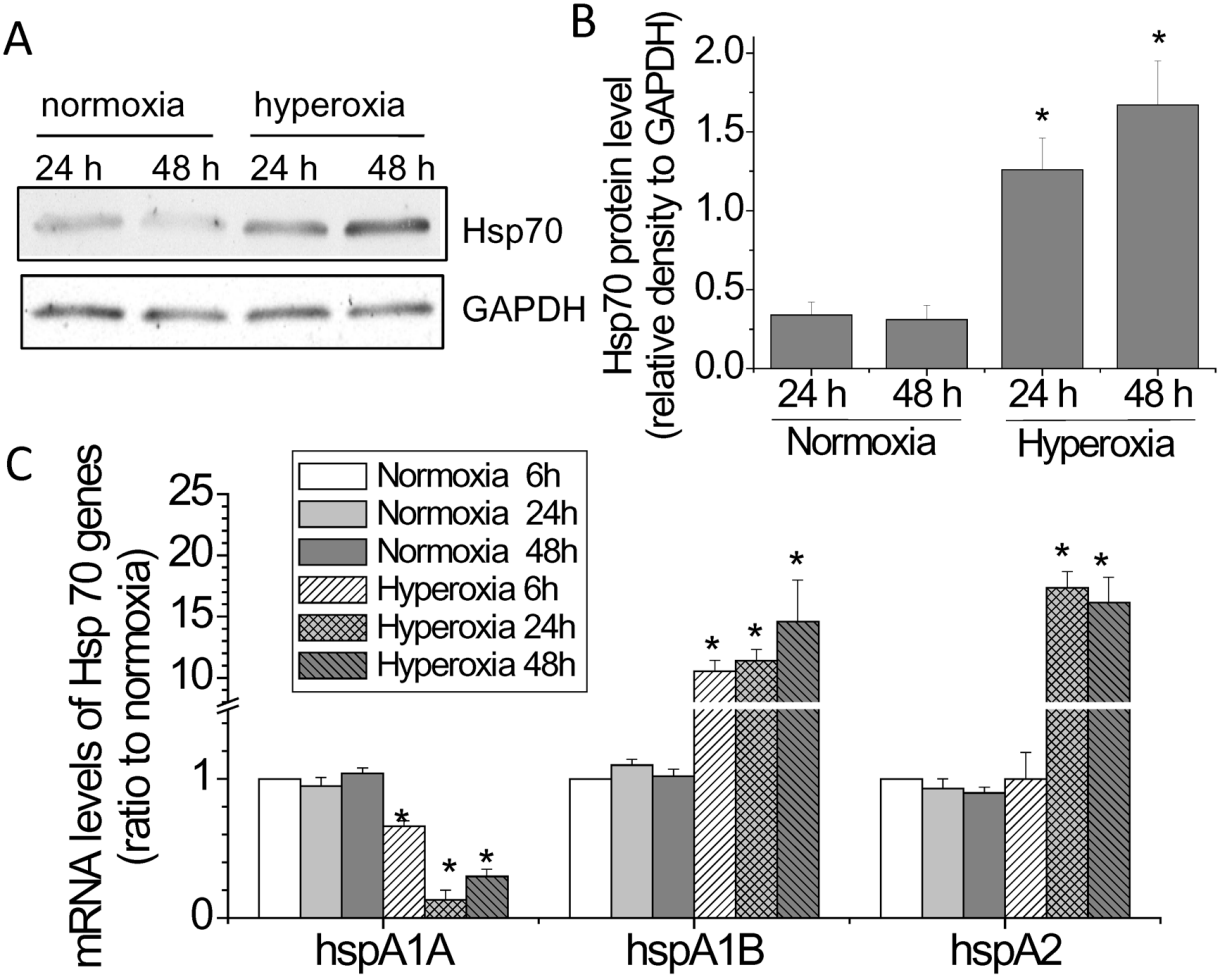
Hyperoxia increases Hsp70 expression. PAECs were exposed to normoxia (room air, 5% CO_2_) or to hyperoxia (95% oxygen, 5% CO_2_) for 1 to 24 h or 48 h after which the protein and mRNA levels of Hsp70 were measured. (A) Representative immunoblots of Hsp70. (B) Bar graph show the changes in Hsp70 protein levels quantified by scanning densitometry. (C) Bar graph showing the changes in the mRNA levels of hspA1A, hspA1B and hspA2 quantified by quantitative real-time PCR. Results are expressed as mean ± SE; n = 4. *P<0.05 vs. normoxia.

In an endeavor to identify proteins involved in hyperoxic endothelial injury, we found that Hsp70 protein expression was increased in BAECs exposed to 95% oxygen. As shown in Fig 1A and 1C, Hsp70 protein levels started to increase after exposure to hyperoxia for 24 h and kept elevated after 48 h. Interestingly, the mRNA expressions of two Hsp70 genes, hspA1B and hspA2, were increased during hyperoxia and the mRNA expression of another Hsp70 gene, hspA1A, was decreased (Fig 1C and 1D), suggesting that the increased Hsp70 protein in hyperoxic PAECs is not from hspA1A but from hspA1B or hspA2.

### Hsp70 inhibitor KNK437exaggerates hyperoxia-induced lung endothelial barrier disruption

**Fig 2:**
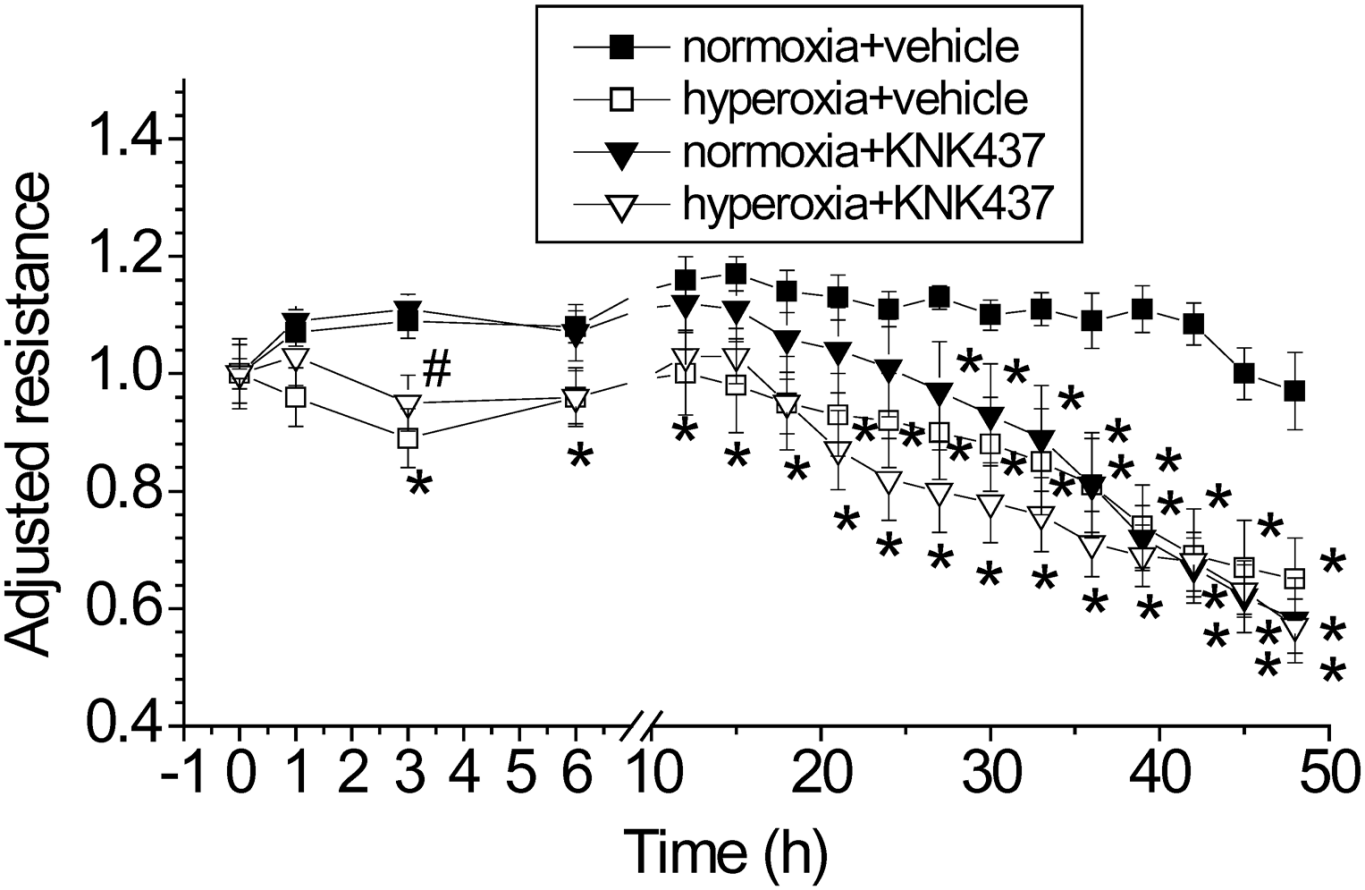
Hsp70 inhibitor KNK437exaggerates hyperoxia-induced lung endothelial barrier disruption. PAECs were treated with and without KNK437 (50 μM) and exposed to normoxia and hyperoxia for 48 h. TEER was continuously monitored as described in Materials and Methods. Results are expressed as mean ± SE; n = 4. * *P*<0.05 vs normoxia+vehicle.

We have reported that hyperoxia induces a two-phase disruption of lung endothelial barrier integrity [4]. In the first phase, the TEER started to decrease at 1 h and reached its lowest at 3 h. In the second phase, the TEER began to decrease at 24 h after hyperoxic exposure and was irreversible and accompanied with endothelial apoptosis [4]. To investigate the role of Hsp70 in hyperoxic lung injury, PAECs were exposed to 95% oxygen in the absence and presence of the Hsp70 inhibitor KNK437. As shown in Fig 2, incubation of PAECs with KNK437 did not affect the first phase but potentiated the second phase disruption of lung endothelial integrity. Because prolonged KNK437 treatment for more than 30 h has maximally increased endothelial permeability, exposure to hyperoxia did not further increase endothelial permeability. These results suggest that Hsp70 may play a protective role in hyperoxic lung endothelial injury.

### Overexpression of Hsp70 protects against hyperoxia-induced lung endothelial barrier disruption

**Fig 3:**
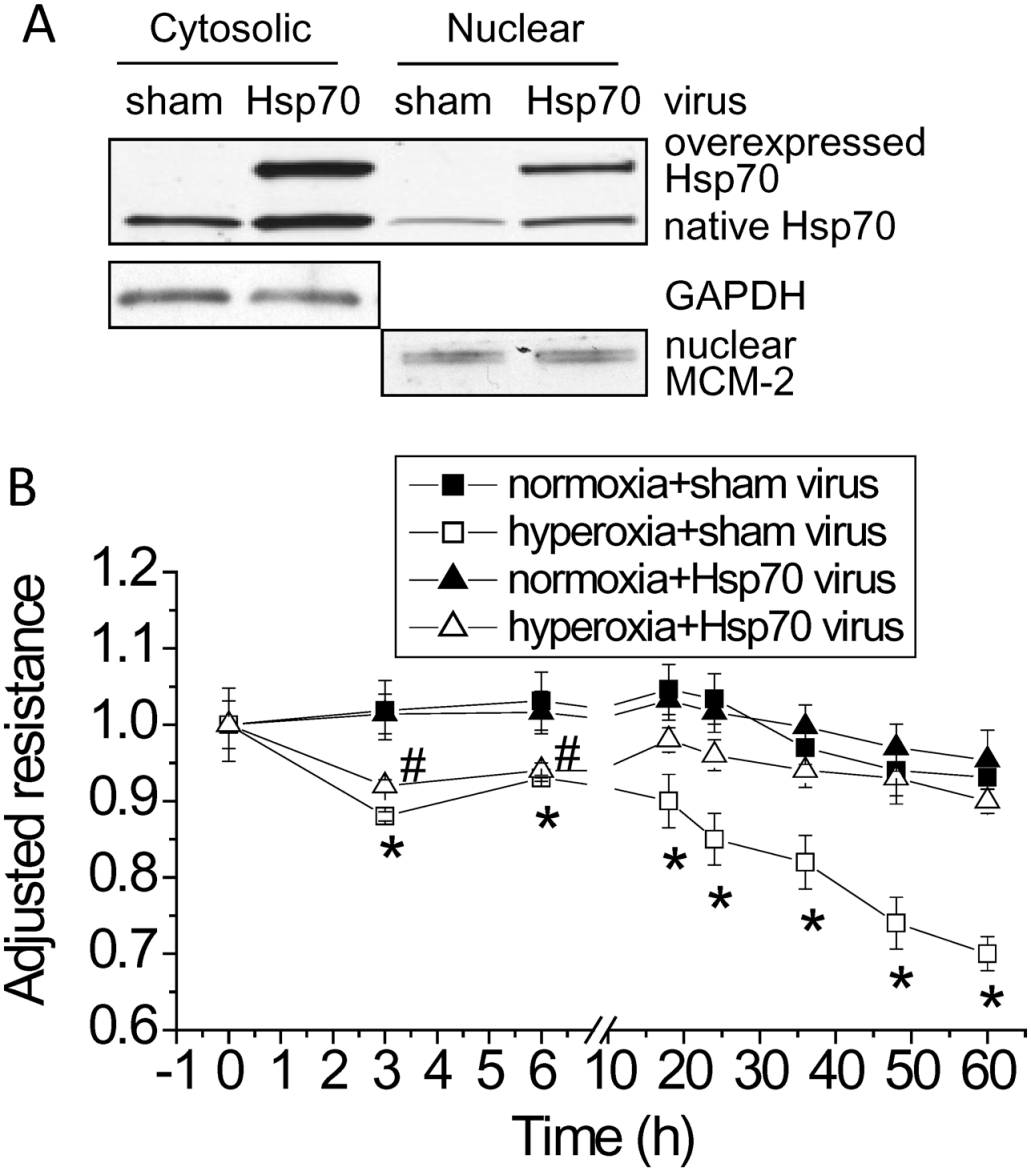
Overexpression of Hsp70 protects against hyperoxia-induced lung endothelial barrier disruption. PAECs were transduced with sham adenovirus or adenovirus containing Hsp70 gene. After incubation for 48 h, cells were then exposed to hyperoxia or normoxia for 60 h. TEER was continuously monitored as described in Materials and Methods. Results are expressed as mean ± SE; n = 3. * *P*<0.05 vs normoxia+sham virus.

To further confirm the role of Hsp70 in hyperoxic lung endothelial injury, Hsp70 was overexpressed using adenovirus containing human hspA1A gene, which encodes human Hsp70 protein. As shown in Fig 3, the first phase of lung endothelial barrier disruption was not affected in PAECs transduced with adenovirus containing Hsp70 gene, but the second phase disruption of lung endothelial integrity was prevented in hyperoxic PAECs conduced with adenovirus containing Hsp70 gene. These data provide further evidence that increased Hsp70 in hyperoxic PAECs protects against hyperoxia-induced disruption of lung endothelial barrier.

### Apoptosis contributes hyperoxia-induced disruption of lung endothelial barrier

**Fig 4:**
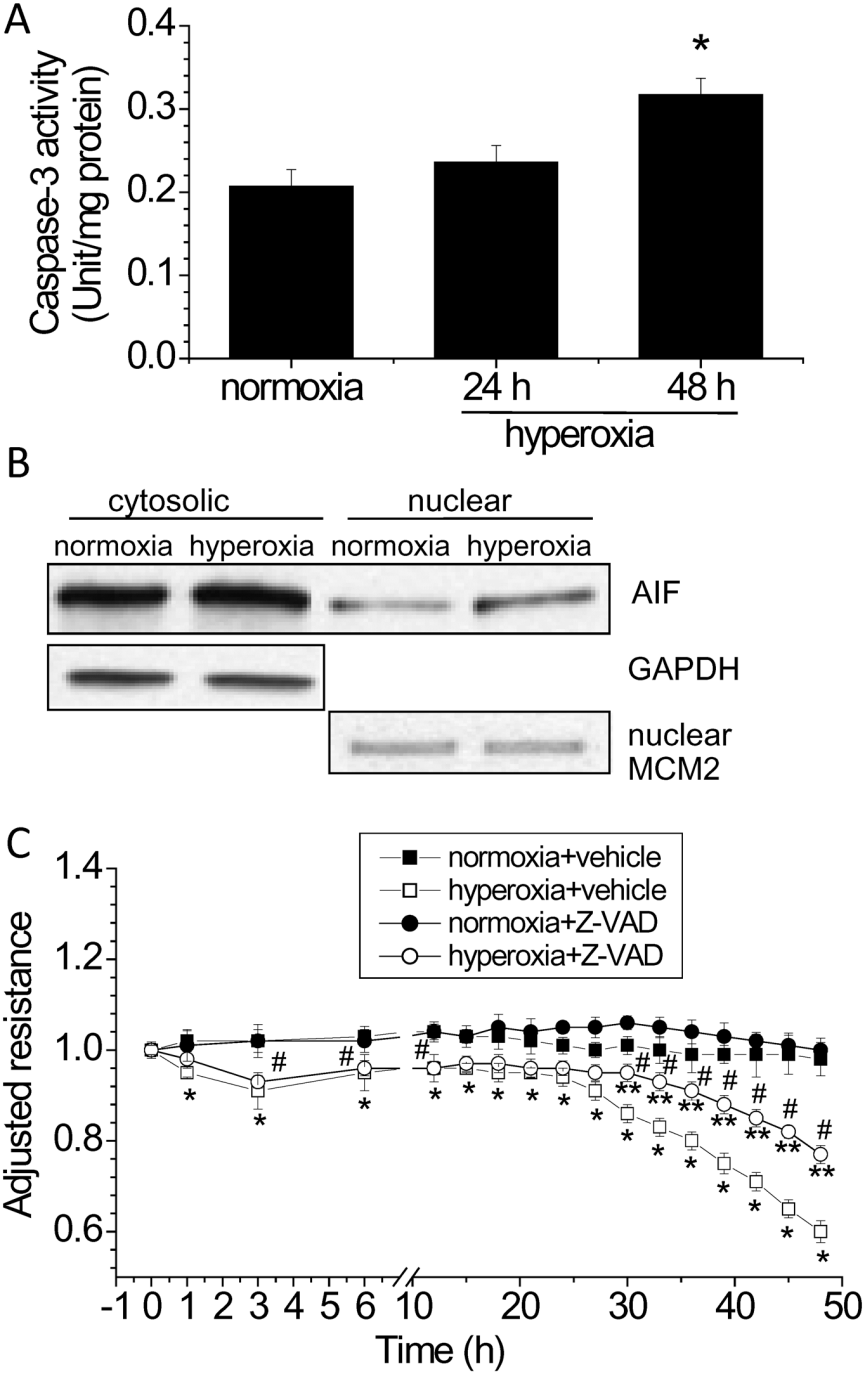
Apoptosis contributes to hyperoxia-induced disruption of lung endothelial barrier. (A and B) PAECs were exposed to normoxia or to hyperoxia for 24 to 48 h after which caspase-3 activity (A) and nuclear AIF protein level (B) was assayed. (C) PAECs were treated with and without the caspase-3 inhibitor Z-VAD-FMK (50 μM) and exposed to normoxia and hyperoxia for 48 h. TEER was continuously monitored as described in Materials and Methods. Results are expressed as mean ± SE; n = 4. * *P*<0.05 vs normoxia; **P<0.05 vs. hyperoxia+vehicle; #*P*<0.05 vs. normoxia+Z-VAD.

Because the second phase of lung endothelial barrier disruption was accompanied with endothelial apoptosis, we investigate whether apoptotic pathways contribute to the disruption of lung endothelial integrity. As shown in Fig 4A and 4B, caspase-3 activity and apoptosis-inducing factor (AIF) in the nuclear fraction were significantly increased in lung endothelial cells exposed to hyperoxia for 48 h, indicating that both caspase-dependent and-independent apoptotic pathways participate hyperoxia-induced disruption of lung endothelial integrity. Moreover, the caspase-3 inhibitor Z-VAD-FMK partially prevented hyperoxia-induced lung endothelial barrier disruption (Fig 4C), suggesting that hyperoxia-induced disruption of endothelial monolayer integrity is due to increased endothelial apoptosis via caspase-3 pathway in part and that caspase-independent pathway, i.e., AIF, may also contribute to endothelial apoptosis of hyperoxic PAECs.

### Inhibition of Hsp70 by using KNK437and Hsp70 siRNA exaggerates hyperoxia-induced endothelial apoptosis and the increases in caspase-3 activity and nuclear AIF protein level

**Fig 5:**
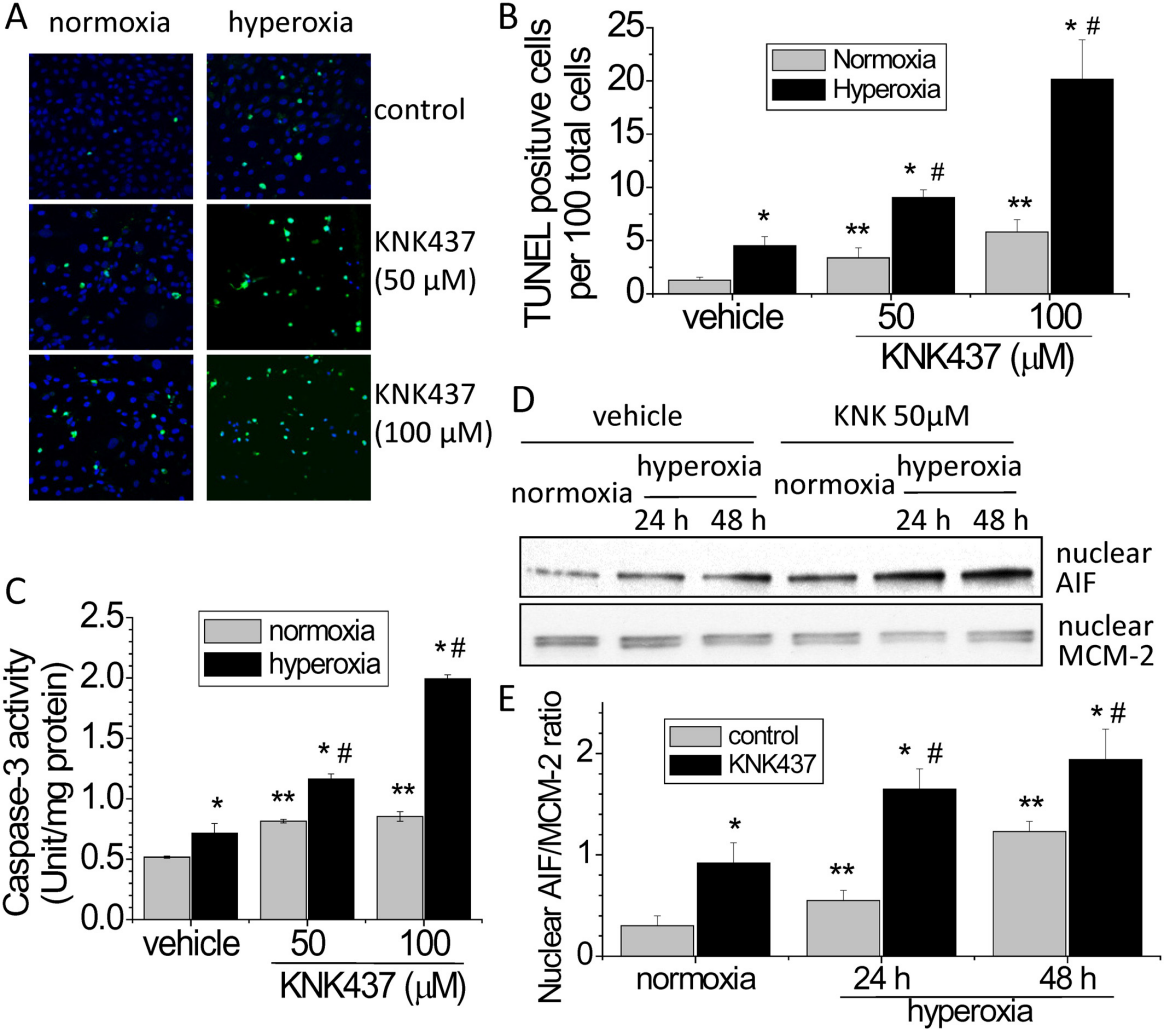
KNK437 exaggerates hyperoxia-induced endothelial apoptosis and the increases in caspase-3 activity and nuclear AIF protein level. PAECs were exposed to normoxia and hyperoxia in the absence and presence of KNK437 (50–100 μM) for 48 h after which TUNEL staining, caspase-3 activity and nuclear AIF protein level were determined. (A) Representative images of TUNEL staining of PAECs exposed to hyperoxia and/or KNK437 for 48 h. (B) Bar graph depicting the changes in the numbers of TUNEL-positive cells. (C) Changes in caspase-3 activity. (D) Representative immunoblots of AIF. (C) Bar graph depicting the changes in nuclear AIF protein levels. Results are expressed as mean ± SE; n = 4. *P<0.05 vs. normoxia; **P<0.05 vs. normoxia+vehicle; #*P*<0.05 vs. hyperoxia+vehicle.

**Fig 6:**
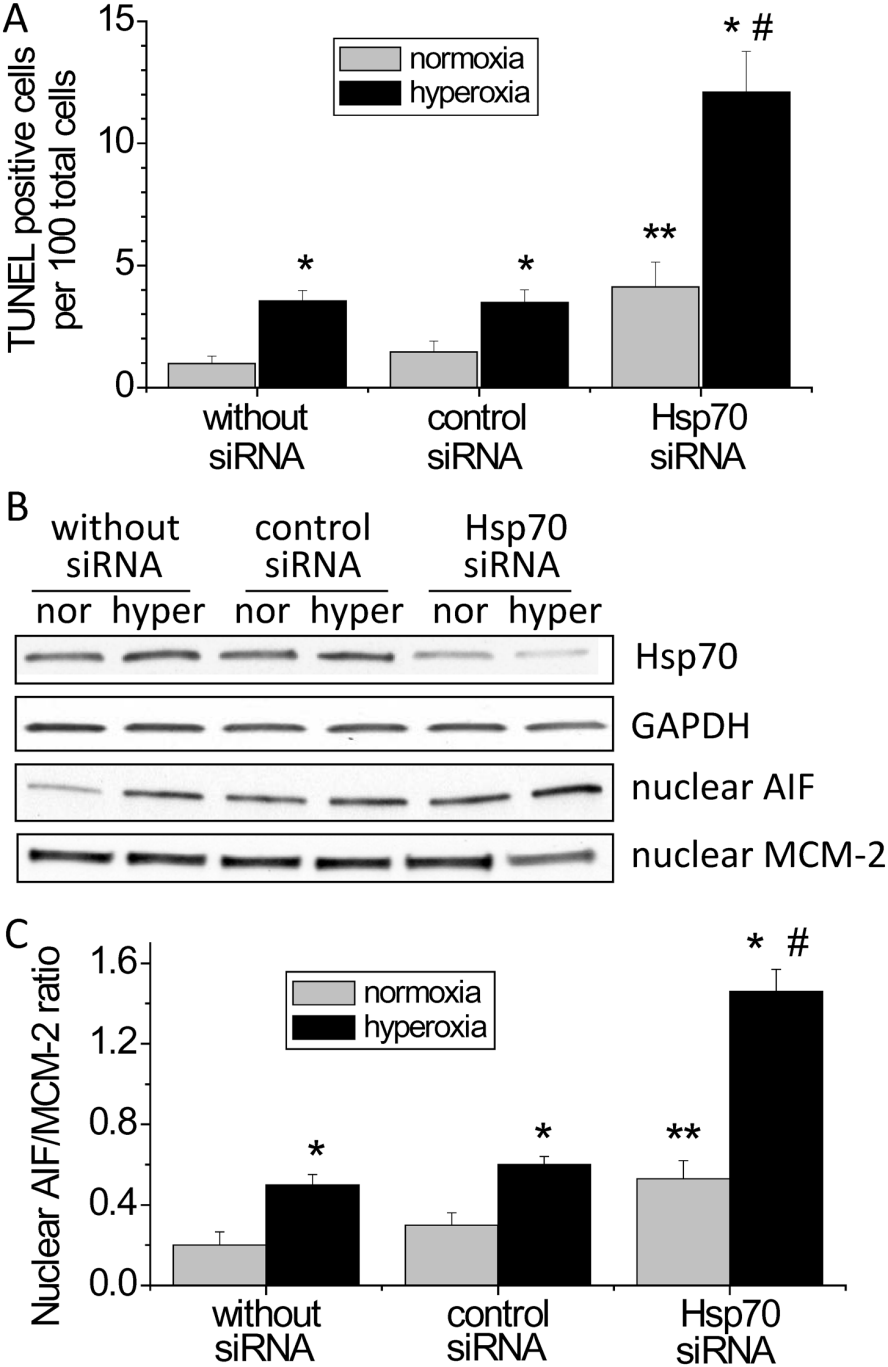
Knocking down Hsp70 exaggerates hyperoxia-induced endothelial apoptosis and the increase in nuclear AIF protein level. PAECs were transfected with control siRNA or siRNA against Hsp70 mRNA (hspA1A and hspA1B) and then exposed to normoxia (nor) and hyperoxia (hyper) for 48 h after which TUNEL staining and nuclear AIF protein level were assayed. (A) Changes in the numbers of TUNEL-positive cells. (B) Representative immunoblots of AIF and Hsp70. (C) Bar graph depicting the changes in Hsp70 and nuclear AIF protein levels. Results are expressed as mean ± SE; n = 4. *P<0.05 vs. normoxia. **P< 0.05 vs. normoxia+control siRNA; #*P*<0.05 vs. hyperoxia+control siRNA.

To investigate the mechanism responsible for protective effect of Hsp70 in hyperoxic lung endothelial injury, we studied the changes in apoptosis, caspase-3 activity and nuclear AIF level in hyperoxic PAECs treated with the Hsp70 inhibitor KNK437 and Hsp70 siRNA. As shown in Fig 5A and 5B, incubation of PAECs with KNK437 resulted in an increase in TUNEL-positive cells in hyperoxic PAECs, suggesting that the protective effect of Hsp70 on hyperoxia-induced disruption of lung endothelial barrier might be caused by inhibiting endothelial apoptosis. More specifically, KNK437 exaggerated hyperoxia-induced increases in caspase-3 activity and nuclear AIF level (Fig 5C, 5D and 5E). Notably, KNK437 has been shown to inhibit the expression of other heat shock proteins such as Hsp27, Hsp40 and Hsp105 [16]. To rule out the non-specific effects of KNK437, the expression of Hsp70 was specifically silenced using siRNA. As shown in Fig 6, the increases in TUNEL-positive cells and nuclear AIF level were much higher in hyperoxic PAECs transfected with Hsp70 siRNA than those with or without control siRNA. These data indicate that Hsp70 protects PAECs from hyperoxia-induced apoptosis via both caspase- and AIF-dependent pathways.

### Overexpression of Hsp70 prevents hyperoxia-induced endothelial apoptosis and the increases in caspase-3 activity and nuclear AIF protein level

**Fig 7:**
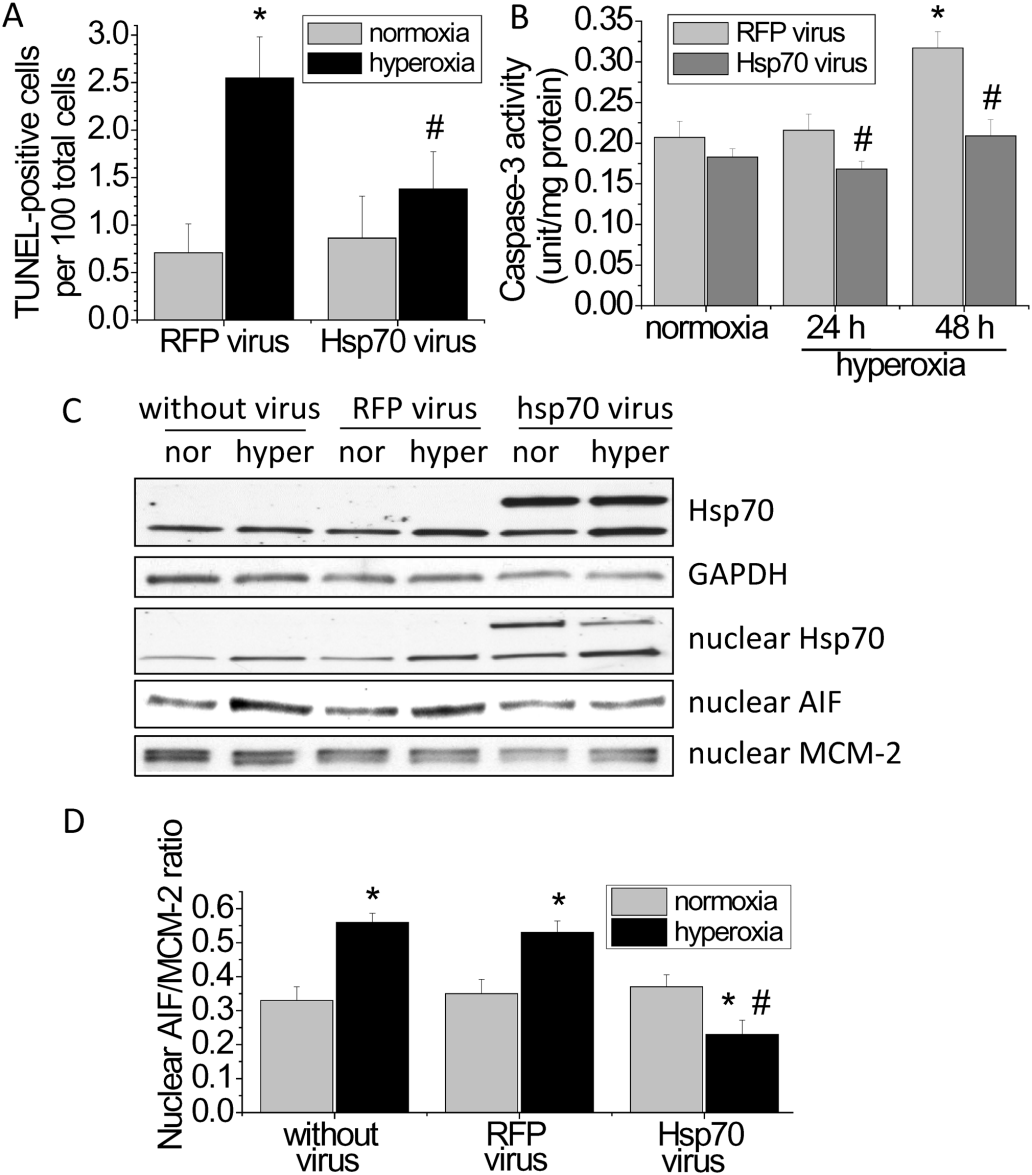
Overexpression of Hsp70 prevents hyperoxia-induced endothelial apoptosis and the increases in caspase-3 activity and nuclear AIF protein level. PAECs were transduced with sham (RFP) adenovirus or adenovirus containing Hsp70 gene. After incubation for 48 h, cells were then exposed to normoxia (nor) and hyperoxia (hyper) for 48 h after which TUNEL staining, caspase-3 activity and nuclear AIF protein level were determined. (A) Changes in the numbers of TUNEL-positive cells. (B) Changes in caspase-3 activity. (C) Representative immunoblots of Hsp70 and AIF. (D) Bar graph depicting the changes in Hsp70 and nuclear AIF protein levels. Results are expressed as mean ± SE; n = 3. *P<0.05 vs. normoxia; #*P*<0.05 vs. hyperoxia+RFP virus.

To further investigate the role of Hsp70 in hyperoxia-induced apoptosis, Hsp70 was overexpressed using adenovirus containing human Hsp70 gene. As shown in Fig 7A, the number of TUNEL-positive cells was reduced in hyperoxic PAECs transduced with adenovirus containing Hsp70 gene than those without Hsp70 gene. More importantly, the increases in caspase-3 activity and nuclear AIF level were prevented in hyperoxic PAECs with Hsp70 overexpression (Fig 7B, 7C and 7D). These data further support that the protective effect of Hsp70 on hyperoxia-induced disruption of lung endothelial barrier is caused by inhibiting the caspase- and AIF-dependent apoptosis.

### Interaction of Hsp70 and AIF in hyperoxic PAECs

**Fig 8:**
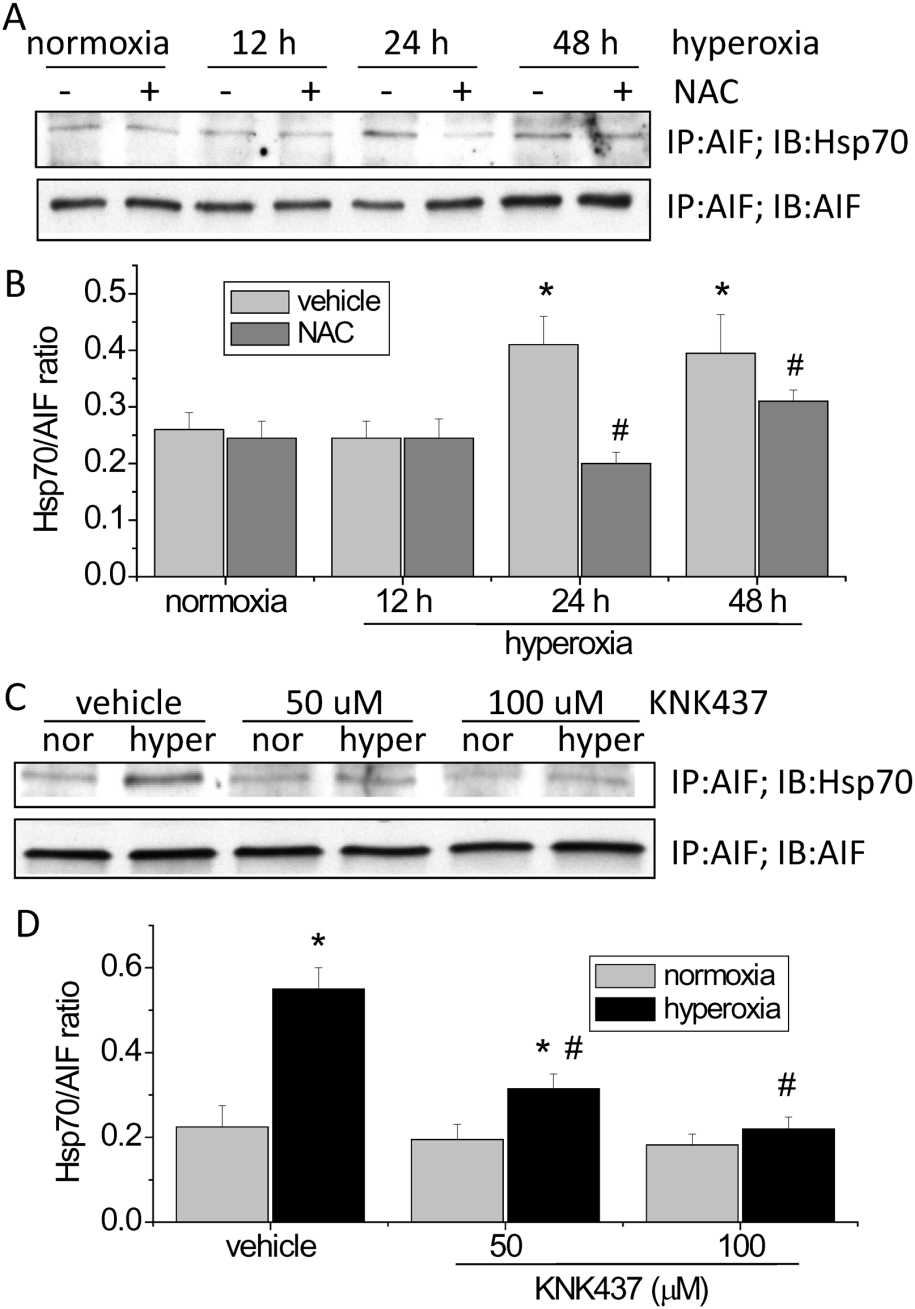
Hsp70/AIF interaction and the effects of NAC and KNK437 on the Hsp70/AIF interaction in hyperoxic PAECs. PAECs were exposed to normoxia (nor) and hyperoxia (hyper) in the absence and presence of NAC (5 mM) or KNK437 (50–100 μM) for 48 h after which co-immunoprecipitations of Hsp70 and AIF in the cytosolic fraction of cell lysates were performed. (A and C) Representative immunoblots of Hsp70 and AIF. (B and D) Bar graph depicting the changes in Hsp70/AIF ratio. Results are expressed as mean ± SE; n = 4. *P<0.05 vs. normoxia; #*P*<0.05 vs. hyperoxia+vehicle.

To study the mechanism for Hsp70-mediated inhibition of AIF nuclear translocation in hyperoxic PAECs, we examined Hsp70/AIF interaction in the cytosolic fraction of cell lysates of hyperoxic PAEC by using co-immunoprecipitation. As shown in Fig 8, the amounts of Hsp70 pulled down by immunoprecipitation of AIF were much higher in hyperoxic PAECs than normoxic cells. Moreover, incubation of PAECs with the ROS scavenger NAC and the Hsp70 inhibitor KNK437 reduced the amounts of Hsp70 pulled down by immunoprecipitation of AIF in hyperoxic PAECs (Fig 8). Inhibition of Hsp70/AIF association by KNK437 correlated with increased endothelial apoptosis and nuclear AIF level in KNK437-treated PAECs. These results suggest that association of Hsp70 with AIF may contribute to the protective effect of Hsp70 on hyperoxia-induced endothelial apoptosis via inhibiting AIF nuclear translocation.

### The hyperoxia-induced increase in Hsp70 expression is ROS-dependent

**Fig 9:**
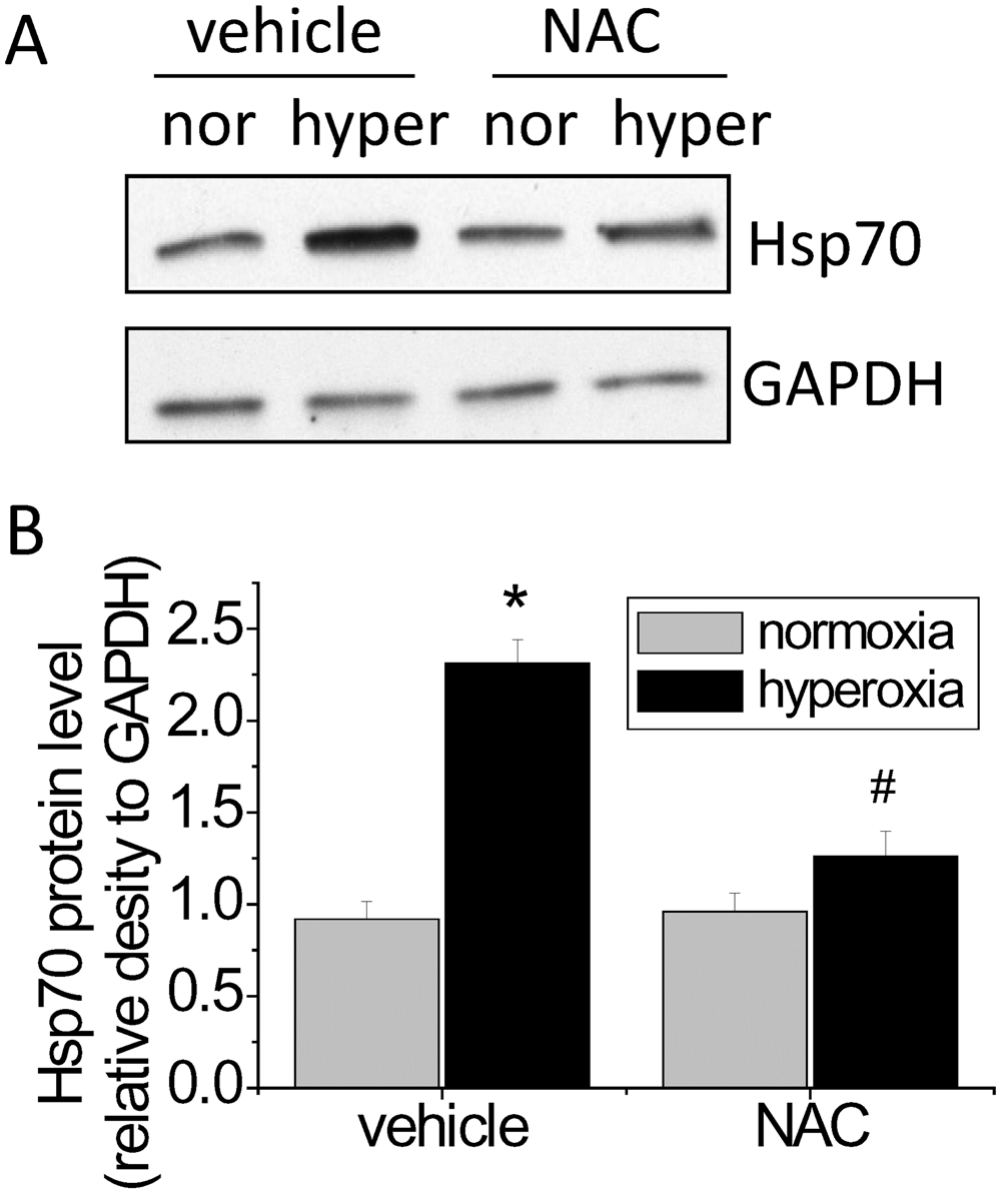
Hyperoxia-induced increase in Hsp70 expression is ROS-dependent. PAECs were treated with and without NAC (5 mM) and exposed to normoxia (nor) and hyperoxia (hyper) for 48 h after which Hsp70 protein level was measured. (A) Representative immunoblots of Hsp70. (B) Bar graph show the changes in Hsp70 protein levels quantified by scanning densitometry. Results are expressed as mean ± SE; n = 4. *P<0.05 vs. normoxia; #*P*<0.05 vs. hyperoxia+vehicle.

We reported that hyperoxia exposure results in burst of ROS production in oxygen toxicity [8,17]. To investigate whether the hyperoxia-induced increase in Hsp70 expression is ROS-dependent, PAECs were exposed to normoxia and hyperoxia in the absence and presence of the ROS scavenger NAC. We found that incubation of PAECs with NAC reduced the increase in Hsp70 protein level in hyperoxic PAECs (Fig 9), suggestion that the hyperoxia-induced increase in Hsp70 expression is due to ROS formation.

## Discussion

The major finding in this study is that hyperoxia induces an increase in Hsp70 expression. Through the experiments of loss and gain of function, we have demonstrated that Hsp70 plays a protective role against endothelial barrier disruption and lung endothelial apoptosis via both caspase- and AIF-dependent mechanism in oxygen toxicity.

Hsp70 protein is encoded by three very closely related paralog genes: hspA1A, hspA1B, and hspA2 [18]. Our data show that the mRNA expressions of hspA1A, hspA1B, and hspA2 are differentially regulated by hyperoxia. Upon exposure to hyperoxia, the expression of hspA1A gene is inhibited. However, the expressions of hspA1B and hspA2 are dramatically stimulated in PAECs exposed to hyperoxia that occurs as earlier as 6–24 h after exposure. Notably, Hsp70 protein expression in hyperoxic PAECs is inhibited by using a mixture of siRNAs against the mRNAs of hspA1A and hspA1B. Thus, we demonstrate, for the first time, that the hyperoxia-induced Hsp70 protein expression is from hspA1B gene. The hyperoxia-response elements in the promotors of hspA1A, hspA1B, and hspA2 might be different though they encode same Hsp70 protein.

Prolonged exposure of PAECs to hyperoxia results in a two-phase barrier disruption of endothelial monolayer [4]. In the first phase, the endothelial barrier dysfunction starts at 1 h and reached lowest at 3 h after starting hyperoxic exposure. The second phase barrier disruption of endothelial monolayer occurs after hyperoxic exposure for 24 h and is attributable to endothelial apoptosis. Neither inhibition nor overexpression of Hsp70 affects the first phase barrier disruption of endothelial monolayer. Nevertheless, inhibition of Hsp70 by using the Hsp70 inhibitor KNK437 or knock down Hsp70 using siRNA exaggerates and overexpression of Hsp70 prevents the second phase disruption of lung endothelial integrity. Moreover, inhibition of Hsp70 exacerbates and overexpression of Hsp70 prevents hyperoxia-induced endothelial apoptosis. Taken together, these data provide solid evidence showing that increased expression of Hsp70 plays a protective role against hyperoxia-induced lung endothelial barrier disruption.

Zhang et al. recently reported that hyperoxia induces an increase in the secretion of Hsp70 from PAECs [19]. Extracellular Hsp70 exert its cytoprotective effect in lung endothelium through toll-like receptor 4 (TLR4) [19]. However, we did not observe increased Hsp70 in the medium of PAECs exposed to hyperoxia (data not shown). It might be possible that Hsp70 interacts to TLR4 prior to secretion into extracellular space. Hyperoxia-induced endothelial apoptosis occurs in caspase-dependent and-independent fashion [4]. Chromatin condensation and DNA degradation triggered by AIF represents the most important caspase-independent apoptotic pathway [20]. We found that caspase-3 activity is increased and AIF protein level in nuclei is significantly higher in PAECs exposed to hyperoxia. Inhibition of Hsp70 enhances apoptosis, caspase-3 activity and intra-nuclear AIF protein levels in hyperoxic PAECs. Further, Hsp70 overexpression inhibits hyperoxia-induced increases in the TUNEL-positive cells, caspase-3 activity, and intra-nuclear AIF protein levels. Thus, the protective effect of Hsp70 on hyperoxia-induced disruption of lung endothelial barrier is caused by inhibiting the caspase- and AIF-dependent apoptosis.

In the caspase-dependent apoptotic pathway, cytochrome c is first released from mitochondria and recruits apoptotic protease activating factor 1 (Apaf-1) and dATP/ATP into an apoptosome complex [21]. This complex then cleaves and activates procaspase-9 and eventually induces apoptosis via caspase-3 activation [21,22]. It has been shown that Hsp70 induces a conformational change of procaspase-9 which blocks the recruitment of procaspase-9 to the Apaf-1/dATP/cytochrome c apoptosome complex leading to inhibition of caspase-3 activity [23]. However, the mechanism for Hsp70-induced inhibition of AIF apoptotic pathway remains unknown. AIF is located in mitochondrial membrane and is activated through calpain-1 cleavage in caspase-independent apoptosis. After being truncated and activated, AIF is released into cytoplasm and translocated to the nuclei where it induces chromatin condensation and DNA degradation [24–26]. We found that Hsp70 interacts with and sequestrate AIF in the cytosol in hyperoxic PAECs. Inhibition of Hsp70/AIF association by KNK437 correlated with increased nuclear AIF level and endothelial apoptosis in KNK437-treated PAECs. These data suggest that association of Hsp70 with AIF contributes to the protective effect of Hsp70 on hyperoxia-induced endothelial apoptosis via inhibiting AIF nuclear translocation. Several mechanisms might be involved in the increase in Hsp70/AIF association in hyperoxic PAECs. First, hyperoxia increases the expression of Hsp70 which binds more AIF protein. Second, hypoxia may induce posttranslational modification of Hsp70 such as phosphorylation and acetylation, which increase the affinity of Hsp70 to AIF.

The mechanism for hyperoxia-induced Hsp70 expression remains unknown. It has been shown that the expression of Hsp70 correlates with ROS production [27,28]. Prolonged exposure of PAECs to hyperoxia leads to elevated ROS and reactive nitrogen species production [4,8,29]. To investigate whether ROS are responsible for Hsp70 expression in hyperoxic PAECs, we determined the effects of the ROS scavenger NAC on Hsp70 protein level in PAECs exposed to hyperoxia. Our data show that scavenging ROS prevents the hyperoxia-induced increase in Hsp70 expression. Moreover, incubation of PAECs with NAC reduces the interaction of Hsp70 with AIF in hyperoxic PAECs. Together, our data indicate that the hyperoxia-induced increase in Hsp70 expression and Hsp70/AIF interaction are contributable to ROS formation. Therefore, ROS not only cause lung endothelial barrier disruption and cell death but also induce various protective mechanisms to prevent cells from hyperoxia-induced damage.

**Fig 10:**
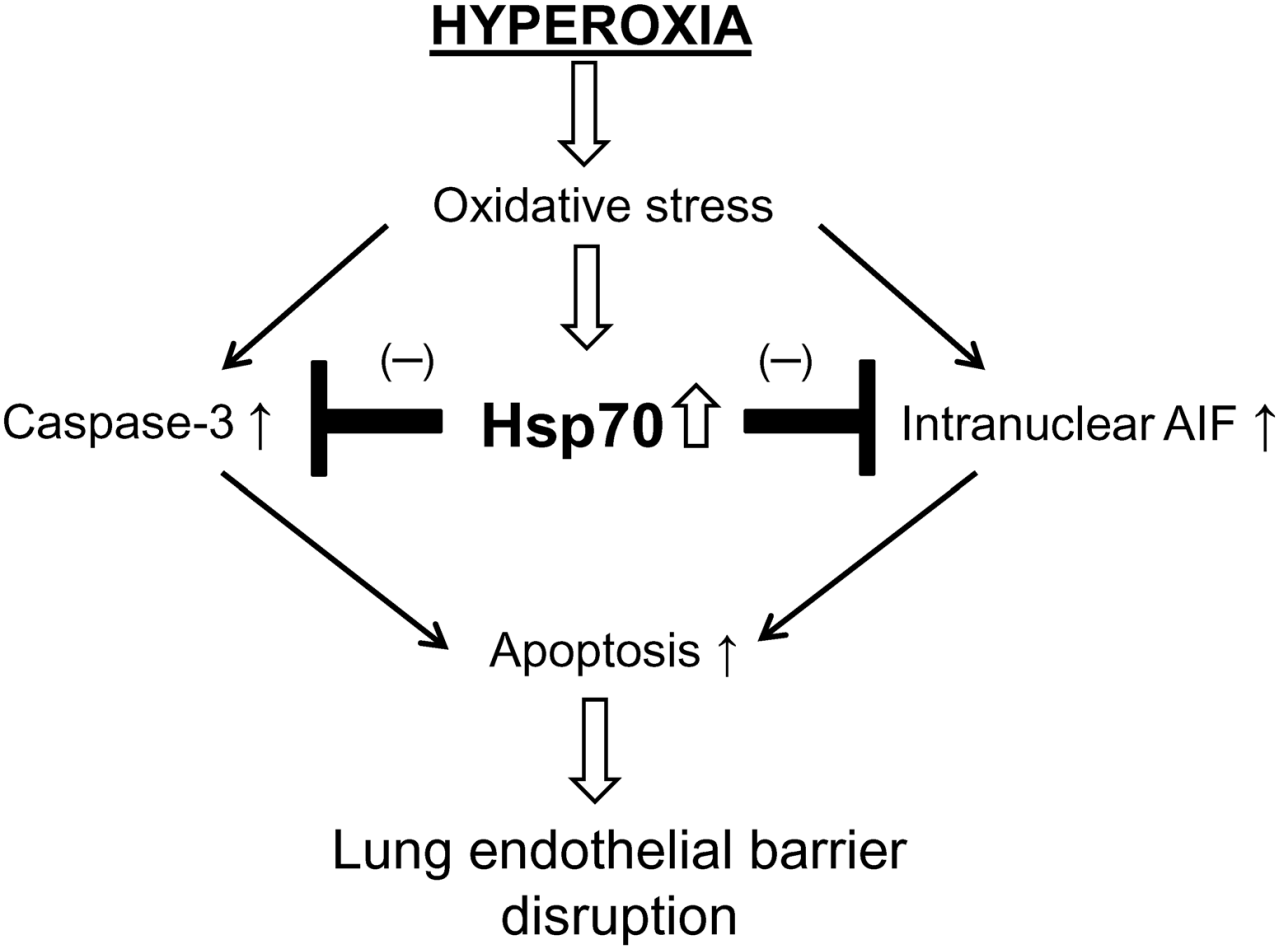
A schematic pathway illustrating the role of Hsp70 in hyperoxic lung endothelial barrier disruption. Hyperoxia induces an increase in Hsp70 expression which plays a protective role against endothelial barrier disruption and lung endothelial apoptosis via caspase- and AIF-dependent mechanism in hyperoxic lung endothelial injury.

In summary, hyperoxia induces an increase in Hsp70 expression that is from hspA1B gene. We have demonstrated that Hsp70 plays a protective role against endothelial barrier disruption and lung endothelial apoptosis via both caspase- and AIF-dependent mechanisms in oxygen toxicity (Fig 10). In addition, association of Hsp70 with AIF prevents AIF nuclear translocation, contributing to the protective effect of Hsp70 on hyperoxia-induced endothelial apoptosis. Manipulation of Hsp70 might be a novel therapy for acute lung injury in oxygen toxicity.

## References

[1] Bhandari V, Elias JA (2006) Cytokines in tolerance to hyperoxia-induced injury in the developing and adult lung. Free Radic Biol Med 41: 4–18. 16781448 10.1016/j.freeradbiomed.2006.01.027

[2] Grath-Morrow SA, Stahl J (2001) Apoptosis in neonatal murine lung exposed to hyperoxia. Am J Respir Cell Mol Biol 25: 150–155. 11509323 10.1165/ajrcmb.25.2.4362

[3] Brasch RC, Berthezene Y, Vexler V, Rosenau W, Clement O, Muhler A et al (1993) Pulmonary oxygen toxicity: demonstration of abnormal capillary permeability using contrast-enhanced MRI. Pediatr Radiol 23: 495–500. 8309747 10.1007/BF02012128

[4] Kondrikov D, Gross C, Black SM, Su Y (2014) Novel peptide for attenuation of hyperoxia-induced disruption of lung endothelial barrier and pulmonary edema via modulating peroxynitrite formation. J Biol Chem 289: 33355–33363. 10.1074/jbc.M114.585356 25315770 PMC4246092

[5] Phillips PG, Tsan MF (1988) Hyperoxia causes increased albumin permeability of cultured endothelial monolayers. J Appl Physiol 64: 1196–1202. 3366737 10.1152/jappl.1988.64.3.1196

[6] Payne DK, Owens MW, Grisham M (1996) Early albumin leakage in pulmonary endothelial monolayers exposed to varying levels of hyperoxia. Free Radic Res 25: 229–238. 8889489 10.3109/10715769609149048

[7] Weir KL, O'Gorman EN, Ross JA, Godden DJ, McKinnon AD, Johnston PW (1994) Lung capillary albumin leak in oxygen toxicity. A quantitative immunocytochemical study. Am J Respir Crit Care Med 150: 784–789. 8087353 10.1164/ajrccm.150.3.8087353

[8] Kondrikov D, Elms S, Fulton D, Su Y (2010) eNOS-beta-actin interaction contributes to increased peroxynitrite formation during hyperoxia in pulmonary artery endothelial cells and mouse lungs. J Biol Chem 285: 35479–35487. 10.1074/jbc.M110.140269 20826796 PMC2975172

[9] Radomski A, Sawicki G, Olson DM, Radomski MW (1998) The role of nitric oxide and metalloproteinases in the pathogenesis of hyperoxia-induced lung injury in newborn rats. Br J Pharmacol 125: 1455–1462. 9884073 10.1038/sj.bjp.0702216 PMC1565728

[10] Azad N, Iyer A, Vallyathan V, Wang L, Castranova V, Stehlik C et al (2010) Role of oxidative/nitrosative stress-mediated Bcl-2 regulation in apoptosis and malignant transformation. Ann N Y Acad Sci 1203: 1–6. 10.1111/j.1749-6632.2010.05608.x 20716276

[11] Vitiello PF, Wu YC, Staversky RJ, O'Reilly MA (2009) p21(Cip1) protects against oxidative stress by suppressing ER-dependent activation of mitochondrial death pathways. Free Radic Biol Med 46: 33–41. 10.1016/j.freeradbiomed.2008.09.022 18948188 PMC2631574

[12] Alphonse RS, Vadivel A, Coltan L, Eaton F, Barr AJ, Dyck JR et al (2011) Activation of Akt protects alveoli from neonatal oxygen-induced lung injury. Am J Respir Cell Mol Biol 44: 146–154. 10.1165/rcmb.2009-0182OC 20348209

[13] Kiang JG, Tsokos GC (1998) Heat shock protein 70 kDa: molecular biology, biochemistry, and physiology. Pharmacol Ther 80: 183–201. 9839771 10.1016/s0163-7258(98)00028-x

[14] Mayer MP (2013) Hsp70 chaperone dynamics and molecular mechanism. Trends Biochem Sci 38: 507–514. 10.1016/j.tibs.2013.08.001 24012426

[15] Chen F, Yu Y, Qian J, Wang Y, Cheng B, Dimitropoulou C et al (2012) Opposing actions of heat shock protein 90 and 70 regulate nicotinamide adenine dinucleotide phosphate oxidase stability and reactive oxygen species production. Arterioscler Thromb Vasc Biol 32: 2989–2999. 10.1161/ATVBAHA.112.300361 23023377 PMC3499642

[16] Yokota S, Kitahara M, Nagata K (2000) Benzylidene lactam compound, KNK437, a novel inhibitor of acquisition of thermotolerance and heat shock protein induction in human colon carcinoma cells. Cancer Res 60: 2942–2948. 10850441

[17] Kondrikov D, Caldwell RB, Dong Z, Su Y (2011) Reactive oxygen species-dependent RhoA activation mediates collagen synthesis in hyperoxic lung fibrosis. Free Radic Biol Med 50: 1689–1698. 10.1016/j.freeradbiomed.2011.03.020 21439370 PMC3097427

[18] Scieglinska D, Piglowski W, Chekan M, Mazurek A, Krawczyk Z (2011) Differential expression of HSPA1 and HSPA2 proteins in human tissues; tissue microarray-based immunohistochemical study. Histochem Cell Biol 135: 337–350. 10.1007/s00418-011-0791-5 21373891 PMC3063884

[19] Zhang Y, Zhang X, Shan P, Hunt CR, Pandita TK, Lee PJ (2013) A protective Hsp70-TLR4 pathway in lethal oxidant lung injury. J Immunol 191: 1393–1403. 10.4049/jimmunol.1300052 23817427 PMC3730854

[20] Delavallee L, Cabon L, Galan-Malo P, Lorenzo HK, Susin SA (2011) AIF-mediated caspase-independent necroptosis: a new chance for targeted therapeutics. IUBMB Life 63: 221–232. 10.1002/iub.432 21438113

[21] Reubold TF, Wohlgemuth S, Eschenburg S (2009) A new model for the transition of APAF-1 from inactive monomer to caspase-activating apoptosome. J Biol Chem 284: 32717–32724. 10.1074/jbc.M109.014027 19801675 PMC2781688

[22] Hu Y, Benedict MA, Ding L, Nunez G (1999) Role of cytochrome c and dATP/ATP hydrolysis in Apaf-1-mediated caspase-9 activation and apoptosis. EMBO J 18: 3586–3595. 10393175 10.1093/emboj/18.13.3586 PMC1171437

[23] Saleh A, Srinivasula SM, Balkir L, Robbins PD, Alnemri ES (2000) Negative regulation of the Apaf-1 apoptosome by Hsp70. Nat Cell Biol 2: 476–483. 10934467 10.1038/35019510

[24] Chen Q, Paillard M, Gomez L, Ross T, Hu Y, Xu A et al (2011) Activation of mitochondrial mu-calpain increases AIF cleavage in cardiac mitochondria during ischemia-reperfusion. Biochem Biophys Res Commun 415: 533–538. 10.1016/j.bbrc.2011.10.037 22057010 PMC3244491

[25] Sevrioukova IF (2011) Apoptosis-inducing factor: structure, function, and redox regulation. Antioxid Redox Signal 14: 2545–2579. 10.1089/ars.2010.3445 20868295 PMC3096518

[26] Cande C, Cohen I, Daugas E, Ravagnan L, Larochette N, Zamzami N et al (2002) Apoptosis-inducing factor (AIF): a novel caspase-independent death effector released from mitochondria. Biochimie 84: 215–222. 12022952 10.1016/s0300-9084(02)01374-3

[27] Sapozhnikov AM, Gusarova GA, Ponomarev ED, Telford WG (2002) Translocation of cytoplasmic HSP70 onto the surface of EL-4 cells during apoptosis. Cell Prolif 35: 193–206. 12153612 10.1046/j.1365-2184.2002.00239.x PMC6495670

[28] Gupta SC, Siddique HR, Mathur N, Vishwakarma AL, Mishra RK, Saxena DK et al (2007) Induction of hsp70, alterations in oxidative stress markers and apoptosis against dichlorvos exposure in transgenic Drosophila melanogaster: modulation by reactive oxygen species. Biochim Biophys Acta 1770: 1382–1394. 17640809 10.1016/j.bbagen.2007.05.010

[29] Usatyuk PV, Singleton PA, Pendyala S, Kalari SK, He D, Gorshkova IA et al (2012) Novel role for non-muscle myosin light chain kinase (MLCK) in hyperoxia-induced recruitment of cytoskeletal proteins, NADPH oxidase activation, and reactive oxygen species generation in lung endothelium. J Biol Chem 287: 9360–9375. 10.1074/jbc.M111.294546 22219181 PMC3308820

